# Non-destructive analysis of sucrose, caffeine and trigonelline on single green coffee beans by hyperspectral imaging

**DOI:** 10.1016/j.foodres.2017.12.031

**Published:** 2018-04

**Authors:** Nicola Caporaso, Martin B. Whitworth, Stephen Grebby, Ian D. Fisk

**Affiliations:** a Campden BRI, Chipping Campden, Gloucestershire, GL55 6LD, UK; b Division of Food Sciences, University of Nottingham, Sutton Bonington Campus, LE12 5RD, UK; c Nottingham Geospatial Institute, Faculty of Engineering, University of Nottingham, Innovation Park, NG7 2TU, UK

**Keywords:** Hyperspectral chemical imaging, NIR chemical mapping, Single seed variability, Coffee sugars, Coffee chemistry, Caffeine

## Abstract

Hyperspectral imaging (HSI) is a novel technology for the food sector that enables rapid non-contact analysis of food materials. HSI was applied for the first time to whole green coffee beans, at a single seed level, for quantitative prediction of sucrose, caffeine and trigonelline content. In addition, the intra-bean distribution of coffee constituents was analysed in Arabica and Robusta coffees on a large sample set from 12 countries, using a total of 260 samples. Individual green coffee beans were scanned by reflectance HSI (980–2500 nm) and then the concentration of sucrose, caffeine and trigonelline analysed with a reference method (HPLC-MS). Quantitative prediction models were subsequently built using Partial Least Squares (PLS) regression. Large variations in sucrose, caffeine and trigonelline were found between different species and origin, but also within beans from the same batch. It was shown that estimation of sucrose content is possible for screening purposes (R^2^ = 0.65; prediction error of ~ 0.7% w/w coffee, with observed range of ~ 6.5%), while the performance of the PLS model was better for caffeine and trigonelline prediction (R^2^ = 0.85 and R^2^ = 0.82, respectively; prediction errors of 0.2 and 0.1%, on a range of 2.3 and 1.1% w/w coffee, respectively). The prediction error is acceptable mainly for laboratory applications, with the potential application to breeding programmes and for screening purposes for the food industry. The spatial distribution of coffee constituents was also successfully visualised for single beans and this enabled mapping of the analytes across the bean structure at single pixel level.

## Highlights

• NIR hyperspectral imaging was applied to green coffee beans on a single seed basis.
• Sucrose, caffeine and trigonelline were analysed from single beans using HPLC-MS.
• PLS regression models were built to quantify for the coffee constituents.
• Prediction models were applied to single pixels to visualise the compound distribution.
• HSI was shown to be useful for rapid non-destructive screening of single coffee beans.

## Graphical abstract

**Figure:**
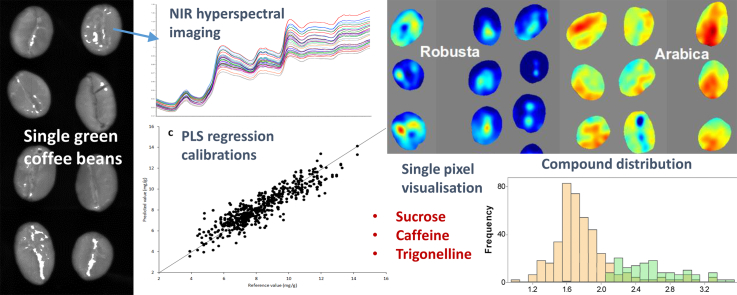

## 1 Introduction

### 1.1 Coffee composition and quality parameters

Coffee is one of the most popular beverages worldwide, and its high commercial value is mainly related to its flavour, which is strictly dependent on the chemical composition of the green coffee beans and their thermal treatment. The level of particular compounds, including sucrose and alkaloids, therefore directly influence the final drinking quality of coffee. The main coffee constituents are carbohydrates (polysaccharides range from 34 to 44% in Arabica and 48–55% in Robusta coffees), followed by lipids, proteins and peptides, and free sugars. The lipid content of coffee is significantly different between Arabica and Robusta, with 15–17% and 7–10% coffee oil for the two species, respectively (Farah, 2012). The sucrose content is reported to have a wide range between batches, from 3.8 to 10.7% (dry weight basis; dwb) based on the analysis of 14 species and 6 new taxa, depending on the botanical and geographical origins (Campa et al., 2004). Green Arabica coffee beans have sucrose content ranging from 6.25 to 8.45%, whereas in Robusta it ranges from 0.9% to 4.85%, with Robusta also containing more reducing sugars (Clarke & Vitzthum, 2008). Other studies have reported sucrose content for Robusta coffee of 4.05–7.05% (dwb) (Ky et al., 2001). The post-harvest processing of coffee beans can also dramatically affect composition; for example, ranges of 2.60–3.02% and 6.60–7.02% have been reported for sucrose in dry-processed and wet-processed green coffee (Clarke & Vitzthum, 2008).

Among the other compounds in coffee, acids and alkaloids both play a critical role in terms of coffee quality, as they influence the flavour of the beverage. Caffeine is a heat stable methylxanthine, with a distinctive bitter taste and a stimulating effect. Caffeine content in Arabica coffee beans is in the range of 0.90–1.3% (Farah, 2012), while it ranges from 1.51 to 3.33% (dwb) for Robusta (Ky et al., 2001). Trigonelline is an alkaloid whose synthesis is carried out by enzymatic methylation of nicotinic acid. Its importance in coffee is mainly related to its degradation during roasting to give several volatile compounds; mainly pyrroles and pyridines. Its concentration in Robusta varies from 0.75 to 1.24% (dwb) (Ky et al., 2001), which is considerably higher than Arabica (Farah, 2012).

Coffee composition is known to vary widely depending on the genotype (Arabica, *Coffea arabica*, or Robusta, *Coffea canephora*), environmental factors such as the geographical origin, and post-harvest processing (Joët et al., 2010). Remarkable differences within the same batch might also be observed, similarly to what has been reported for other crops; e.g. hazelnut plants, which showed significant differences in chemical composition even within the same plant (Pannico et al., 2017).

Trigonelline and sucrose content is dependent on the coffee genotype, with trigonelline content reported to range from 0.39 to 1.77% (dwb) depending on the species (Campa et al., 2004). However, in contrast, some studies have found that sucrose, caffeine and trigonelline concentrations in green coffee seem not to be significantly affected by the country of origin, nor the genetic groups (Ky et al., 2001). Differences in sucrose content are also linked to the degree of ripening, pre and post-harvest processing (Clarke & Vitzthum, 2008), as well as post-harvest processing (Casal, Oliveira, Alves, & Ferreira, 2000).

### 1.2 Destructive methods for coffee composition analysis

The analysis of coffee constituents is usually performed by wet chemistry, particularly high-performance liquid chromatography (HPLC), while caffeine analysis can be also carried out by spectrophotometric measurement of the extracts. HPLC coupled with Diode-Array Detector (DAD) has been applied for the simultaneous analysis of trigonelline, nicotinic acid and caffeine in coffee (Casal, Oliveira, & Ferreira, 1998). More recently, Perrone, Donangelo, and Farah (2008) described a method which also allowed the identification and quantification of sucrose, based on HPLC-MS analysis. However, despite the advances in analytical speed and the possibility of simultaneously analysing sucrose, caffeine and trigonelline with a single coffee extraction, the destructive nature of the testing and the considerable time required for the grinding, extraction and analysis is impractical for industrial settings. For this reason, the development of fast, non-destructive techniques for green coffee composition is of great research and commercial interest. Moreover, the study of single beans without grinding offers the possibility of understanding natural variability on an individual coffee bean basis. This would offer the potential for the selection of breeding lines with desired characteristics, as also suggested for other commodities such as wheat (Caporaso, Whitworth, & Fisk, 2018).

### 1.3 NIR and HSI for non-destructive analysis of coffee

The coffee industry requires rapid, low cost analytical techniques which ideally are non-destructive for the quantification of chemical properties. Near-Infrared Spectroscopy (NIRS) has these advantages, and its potential to analyse bulk coffee properties has been reported by several authors for caffeine (Fox et al., 2013, Pizarro et al., 2007), chlorogenic acids (Martín, Pablos, & González, 1998) and total sugar prediction (dos Santos Scholz et al., 2014), as well as to verify coffee roasting degree (Alessandrini, Romani, Pinnavaia, & Dalla Rosa, 2008). In particular, Pizarro et al. (2007) presented a prediction model based on reflectance NIR in the region 1100–2500 nm for caffeine content prediction in ground and roasted coffee using Partial Least Squares (PLS) regression. Although primarily focused on the analysis of major compounds, Fourier-Transform Infrared (FT-IR) and NIRS based coffee bean quality evaluation has also involved classification according to several parameters. For example, coffee beans have been classified into different classes of defects – namely black, dark sour and light sour – using Linear Discriminant Analysis (LDA), based on correlations with carbohydrate, lipid, protein and caffeine concentrations (Craig Carneireiro, 2013). However, despite numerous NIR spectroscopy-focussed studies of coffee properties, most utilise ground material, so information on the natural variability among coffee beans is lost and no indication of the spatial distribution of chemical compounds within individual beans can be obtained.

Hyperspectral imaging (HSI) integrates NIR spectroscopy with imaging, so that surface chemistry can be evaluated in a rapid and non-destructive way, thus providing information on the spatial distribution of major chemical constituents across a sample (Liu, Zeng, & Sun, 2015). HSI can analyse the spectra at a single pixel level, and can be further advanced using the “push-broom” system for continuous on-line scanning of samples. The application of HSI in food science, and especially for non-processed food materials, is relatively new and its full potential has not been fully explored (Elmasry, Kamruzzaman, Sun, & Allen, 2012). Some NIR calibrations have been developed for single beans, e.g. for caffeine prediction (Fox et al., 2013), but their applicability is limited as the presentation of the sample does not allow the scanning of several beans at a time.

Hyperspectral imaging has been only recently applied for coffee quality screening purposes. Nansen, Singh, Mian, Allison, and Simmons (2016) applied HSI to characterize commercial roasted and ground coffee batches from the market to assess the consistency of their quality. Calvini, Ulrici, and Amigo (2015) presented a case study of HSI on coffee beans to demonstrate a new sparse method for pixel classification within the hypercube. A recent paper by Cho, Bae, Cho, and Moon (2017) reported on the use of HSI to investigate the qualitative properties of defective roasted coffee beans, which can be considered as different degrees of roast. The authors used one batch only, which was split into five sub-samples and treated under different time-temperature profiles. The classification model built on the spectral region 1000–1700 nm produced accuracies of 85.7% and 86.7% for the calibration and validation datasets, respectively.

Similar studies have used bulk NIR rather than HSI, focussing on ground coffee samples or roasted coffee rather than intact green coffee beans. Accordingly, little is known about the distribution of coffee constituents at single coffee bean level. This can, however, be revealed using HSI, which also offers the advantage of analysing several beans across the same line and provides the opportunity for implementation in the food industry or research laboratories to scan large numbers of samples. Even where HSI has been previously applied to whole coffee beans, the majority of these cases have been for classification without reference measurement and not for quantification purposes. Moreover, this analysis was typically limited to the NIR wavelength region below 1700 nm. To date, limited research has been carried out on seed constituents other than moisture, fat and protein, especially for coffee, despite the advantages offered by NIR technology. The use of HSI in this context would enable non-destructive estimation of the distribution of quality-related bioactive constituents of whole single seeds, which can be used for further analyses or industrial use, in breeding programmes or to obtain a more consistent or improved product. In addition, building prediction models would further allow the use of HSI calibrations to investigate single bean quantitative variation of quality-relevant chemical compounds.

Therefore, the aim of this study is to explore the feasibility of HSI, in the range 980–2500 nm, for the determination of sucrose, caffeine and trigonelline concentration in individual green coffee beans and to predict these compounds using PLS regression. Consequently, these models can be used to visualise the distribution of these compounds at a single pixel level. A large dataset is used to report on the natural variability and distribution of these compounds within and between batches.

## 2 Materials and methods

### 2.1 Coffee samples, chemicals and reagents

Green coffee samples were sourced from several producing locations worldwide, comprising Brazil, Colombia, Costa Rica, Ethiopia, Guatemala, Honduras, India, Kenya, Mexico, Nicaragua, Rwanda, Uganda and Vietnam. These included batches treated using both the washed post-harvest system and the drying system (“natural”), as well as a semi-washed sample from Rwanda and a Monsoon Malabar treated batch from India. Twenty-seven batches were sampled, of which 60% were wet processed and the remaining 40% dried-processed. Ten single beans were randomly sampled from each coffee batch for the single bean experiment. Sucrose, trigonelline and methanoic acid (purity > 95%) were purchased from Sigma-Aldrich (St. Louis, MO, USA), whereas caffeine (98.5% purity) was purchased from Acros organics (New Jersey, USA). Lead (II) acetate basic solution was purchased from VWR International Ltd. (Lutterworth, UK) and HPLC grade methanol was purchased from Fisher scientific (Loughborough, UK). All other chemical and reagents used were of analytical grade.

### 2.2 Hyperspectral NIR imaging analysis

The HSI system used in this study was a line-scanning instrument, referred to as the “push-broom” approach. It enables the scanning of samples while they are moving under a camera that is placed in a fixed position. Each pixel of the obtained “hypercube” (three-dimensional hyperspectral image cube) contains a full spectrum in the range 980–2500 nm. Single green coffee beans were scanned on both sides using an HSI system supplied by Gilden Photonics Ltd. (Glasgow, U.K.), which includes a SWIR spectral camera (Specim Ltd., Oulu, Finland) containing a cooled 14 bit 320 × 256 pixel mercury‑cadmium-telluride (HgCdTe) detector and N25E spectrograph. Therefore, the total number of spectral bands that the detector was able to acquire was 256, but the first 16 bands were removed due to the sensor response. Ten coffee beans were scanned within each hypercube to build calibrations. The samples were placed on a black plastic stage whose motion was controlled by a stepper motor via the software, using a speed that permits the acquisition of appropriate image sizes in the horizontal direction. After a first scan, beans were manually rotated and scanned on the opposite surface. The sample scanning conditions and HSI data treatment were previously reported in detail by Caporaso, Whitworth, and Fisk (2017). In summary, the IDL 8.4 and ENVI 5.2 software (Harris, Florida, USA) was used to process the HSI data and to export mean log(1/R) spectra for each bean. The reflectance spectra of the sample hypercubes were calculated using a white (PTFE material) and a black reference (by automatically closing the camera shutter after each scanning) by applying the following Equation (1):

(1) $$R=\frac{I-D_{s}}{W-D_{w}}\;\frac{t_{w}}{t_{s}}$$

where *R* is the reflectance spectrum, *I* is the recorded spectral intensity at each pixel and spectral band, *W* is the white reference signal, *D*_*s*_ and *D*_*w*_ are the black references (reflectance ~ 0%) for the sample and white reference, respectively, and *t*_*s*_ and *t*_*w*_ are the sample and white exposure times, respectively. The inclusion of *t*_*w*_ and *t*_*s*_ is to account for differences in the exposure times between the white reference and sample measurements. In our case, the exposures used for the white reference and for the sample were different in order to provide optimal dynamic range for the samples. The absorbance spectra, *A*, are then calculated using a logarithm calculation as follows (2):

(2) $$A=\log\;\frac{1}{R}$$

A program written in IDL was applied to perform image segmentation. A region of interest (ROI) was automatically selected for each bean. The average spectra for each coffee bean were then exported for statistical analysis.

### 2.3 Caffeine, sucrose and trigonelline extraction

The reference analysis of caffeine, sucrose and trigonelline was performed according to Perrone et al. (2008). Green coffee beans previously scanned by HSI were ground individually using a Perten 3100 electric grinder (Perten, Hägersten, Sweden). Approximately 0.1 g of the recovered material was accurately weighed; 20 mL boiling distilled water was added, and immediately shaken in a Stuart Orbital incubator SI500 at 250 rpm for 15 min. After cooling the flask under running water, 0.1 mL of a saturated lead acetate solution was added to clarify the extract. Samples were then centrifuged for 5 min at 3000 rpm using a Rotina 380R centrifuge (Hettich, Tuttlingen, Germany). The supernatant, excluding the upper lipid phase, was recovered and filtered through a 0.45 μm Millex MF-Millipore membrane. The final extract was diluted 1:5 with distilled water for HPLC/MS analysis.

### 2.4 HPLC/MS analysis

The coffee extract was analysed using an Agilent 1100 HPLC coupled with a Quattro Ultima MS/MS system, according to Perrone et al. (2008), with slight modifications. Chromatography separation was performed using a Phenomenex Kromasil 5ODS (C18) column (250 mm × 3.2 mm i.d. × 5 μm particle size, Waters, Milford, USA). Eluent A comprised water with 0.3% methanoic acid, while eluent B was methanol. The HPLC program started with 25% B, increased to 65% B after the injection, and was held for 7 min. The ratio was then altered to 25% B at 7 min and held for another 4 min to equilibrate the column. The injection volume was 5 μL and column temperature was 40 °C. The electrospray ionization source operated in negative ion mode during the first 4 min, while the positive ion mode was used from 0 to 7 min. The source temperature was 100 °C, and the desolvation temperature was held at 400 °C. The cone gas flow was 100 L h^− 1^ while the desolvation flow was 500 L h^− 1^. The capillary voltage was set at 2.0 V for ES^+^, and 2.5 V for ES^−^, with a cone voltage of 40 V and 50 V, respectively. The multiplier was set at 500 for ES^+^, and 650 for ES^−^. The detector was operated in Selective Ion Mode using the following m/z for the three compounds: 387 (sucrose), 195 (caffeine) and 138 (trigonelline). Quantification was performed using calibrations established with pure compounds. The coffee extracts were analysed in duplicate. The data were processed in MassLynx 4.0 (Waters, Milford, USA) and Microsoft Excel, and exported for the statistical analysis.

### 2.5 Recovery experiment and limits of detection and quantification

A recovery experiment was performed using a green Mexican Arabica sample as a blank for spiking with the standard compounds. Three different levels of spiking were applied. Triplicate analyses were performed for each level and for the unspiked sample. The limits of detection (LOD) and quantification (LOQ) were calculated as three times and ten times the signal-to-noise ratio (S/N), respectively (Perrone et al., 2008).

### 2.6 Data processing and statistical analysis

Following normalisation of spectral data in IDL to calculate absorbance, four processing operations were performed: bad pixel correction; background removal and objects selection; calculation of mean spectra for each object; and export of the average spectra (Caporaso, Whitworth, & Fisk, 2017). These were analysed to develop calibrations using The Unscrambler X 10.3 software (CAMO, Trondheim, Norway) for statistical analysis. Several spectral pre-processing methods were tested, including the first and second derivative using Savitzky-Golay smoothing (5 points smoothing window, second order polynomial), Multiplicative Scatter Correction (MSC), Standard Normal Variate (SNV), de-trending, etc., before applying a multivariate statistical analysis. A PLS regression was built using the average spectrum obtained from each green coffee bean and the corresponding reference measurements of sucrose, caffeine and trigonelline. This approach was used to build the three different prediction models on the reference data expressed on “as is” basis. To test whether the use of dry matter basis reference data could give a better prediction performance, a previously established moisture calibration (Caporaso, Whitworth, Grebby, & Fisk, 2017) was applied on the hypercubes so that the predicted moisture content was calculated and the reference measurements were also expressed on a predicted dry matter basis. The models were validated using the cross-validation method, randomly selecting 20 subgroups (segments). The best PLS models were chosen according to the regression coefficients, and prediction error of the calibration (RMSEC) and cross-validation (RMSECV) datasets. The optimal number of Latent Variables (LV) was chosen based on plots of RMSECV against number of LVs, to minimise the prediction error while avoiding overfitting (Gowen, Downey, Esquerre, & O'Donnell, 2011).

## 3 Results and discussion

### 3.1 Accuracy of analytical determinations and natural variability of coffee constituents

The statistics for the reference measurements, the results for the recovery experiment and the limits of detection (LOD) and quantification (LOQ) are reported in Table 1. To understand and minimise analytical errors, duplicate HPLC-MS analysis was performed on each extract, on a total of 277 coffee bean samples analysed. The LOD and LOQ of the method were very low with respect to the concentrations of coffee constituents found in the analytes, the LOQ being 26.3, 81.7 and 56.5 mg kg^− 1^ of coffee for caffeine, sucrose and trigonelline, respectively. The recovery experiment resulted in an excellent recovery (≥ 95%) for caffeine and sucrose, and ≥ 80% recovery for trigonelline. These results are in line with previous findings, also regarding the fact that the recovery of some coffee compounds was lower at the higher levels of spiking (Perrone et al., 2008). The results for the reference measurements were acceptable in terms of accuracy of determination and repeatability (average repeatability for sucrose = 3.7%, caffeine = 2.4%, trigonelline = 2.5%).

**Table 1:** Reference measurements on single green coffee beans: a) descriptive statistics, expressed on “as is” basis and on dry weight basis (*n* = 277); b) recovery and repeatability of pure reference compounds; c) the limit of detection (LOD) and limit of quantification (LOQ) obtained by LC/MS analysis.

a	Compound	Mean (mg g^− 1^)	SD (mg g^− 1^)	Min (mg g^− 1^)	Max (mg g^− 1^)
“As is”	Sucrose	43.3	10.2	5.3	70.8
	Caffeine	18.1	4.7	9.2	31.9
	Trigonelline	8.3	2.0	3.9	15.0
dwb	Sucrose	47.8	11.5	5.8	80.0
	Caffeine	19.9	5.1	10.2	34.8
	Trigonelline	9.1	2.2	4.3	17.0


b	Compound	Spike level (mg g^− 1^)	Recovery (%)	Repeatability (%)
	Sucrose	10	80.6 ± 1.6	5.8
		40	111.6 ± 0.2	1.8
		80	94.4 ± 0.2	3.6
	Caffeine	10	112.0 ± 5.3	4.7
		20	104.0 ± 2.8	1.7
		30	91.8 ± 1.9	0.9
	Trigonelline	5	84.9 ± 0.3	1.0
		10	84.0 ± 0.3	1.7
		20	71.0 ± 0.5	4.7


c	Compound	LOD (mg g^− 1^)	LOQ (mg g^− 1^)
	Sucrose	7.9 × 10^− 3^	26.3 × 10^− 3^
	Caffeine	24.5 × 10^− 3^	81.7 × 10^− 3^
	Trigonelline	17.0 × 10^− 3^	56.5 × 10^− 3^

*Values for a) are expressed as mg g^− 1^ of ground coffee material. Values for the recovery are the mean of triplicate analysis, followed by the standard deviation (SD). The recovery experiment was performed on a batch of ground Arabica green coffee beans from Mexico. Repeatability is expressed as relative SD. The LOD and LOQ were calculated according to Perrone et al. (2008).

**Supplementary Figure 1:**
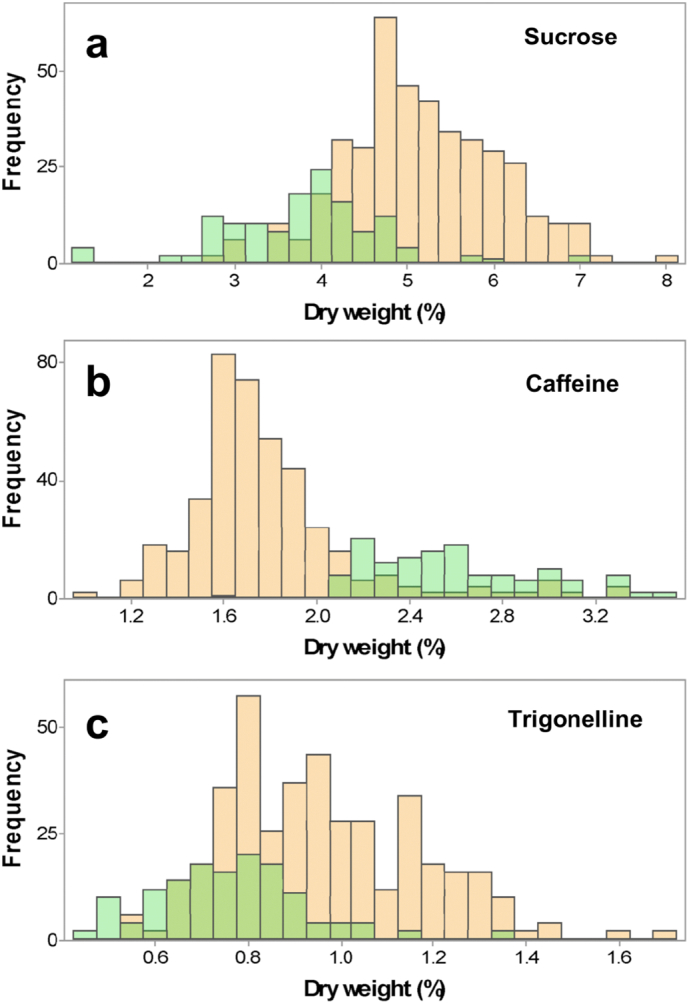
Distribution plots of (a) sucrose, (b) caffeine and (c) trigonelline in single green coffee beans analysed by HPLC/MS, by discriminating Arabica (orange) and Robusta samples (green). The values are expressed as % (dmb).

The ranges of sucrose, caffeine and trigonelline concentration in single green coffee beans are reported in Table 1a. The reference measurements are shown on an “as is” basis, which was the actual analysis performed, as well as on a predicted dry weight basis. All the compounds analysed showed a broad range of concentrations. For example, the maximum sucrose level was over ten times higher than the lowest and in the case of caffeine and trigonelline, the maximum concentrations were three or four times higher than the minimum. As expected, the concentrations of sucrose and trigonelline were significantly higher in Arabica than Robusta coffee beans, while caffeine concentration was higher in Robusta than Arabica coffee. The average sucrose content was 46.7 ± 7.5 mg g^− 1^ for Arabica and 33.6 ± 9.1 mg g^− 1^ for Robusta, while caffeine content was 15.7 ± 2.3 and 19.9 ± 4.9 mg g^− 1^ (“as is”), respectively. A statistically significant difference was observed for all the compounds between Arabica and Robusta (*p* < 0.001), but a region of overlap was observed in the middle region for all three compounds (please refer to Supplementary Figure 1 for further details).

Previous studies investigating the natural variability of sucrose, caffeine and trigonelline in green coffee reported similar average values as the present experiment (Campa et al., 2004, Casal et al., 1998, Dessalegn et al., 2007, Ky et al., 2001). However, the reported standard deviation and the range is usually more narrow, e.g. Dessalegn et al. (2007) studied 42 accessions of Ethiopian Arabica coffees, and reported a standard deviation of 0.82%, 0.1% and 0.13% for sucrose, caffeine and trigonelline, respectively.

The work presented herein also gives information on the variability at a single coffee bean level. A smaller variation would be expected for batch measurements compared to single coffee beans and due to the small sample range. For example, the average batch values of our samples compare favourably with previous works reporting on Arabica coffees (Ky et al., 2001). The variation of sucrose content in green coffee beans is attributed to several factors, including the post-harvest processing and the coffee species, as well as agronomic factors. Robusta coffee showed a higher caffeine content than Arabica beans, although some overlap was observed, especially around 20–25 mg g^− 1^ (dwb). For trigonelline, an opposite trend was found, with Robusta lower in trigonelline than Arabica, i.e. 7.3 ± 1.7 vs 8.6 ± 1.8 mg g^− 1^ (“as is”), respectively. These results are in agreement with the literature, and demonstrate similar overlapping between these species (Campa et al., 2004).

The results of the recovery and repeatability experiment, carried out to verify the performance of the LC/MS method set-up to extract and quantify the coffee constituents in single green coffee beans, is reported in Table 1b. The recovery ranged from 112% for samples spiked with 10 mg g^− 1^ caffeine, to 71% for trigonelline at 20 mg g^− 1^ spiking.

**Supplementary Figure 2:**
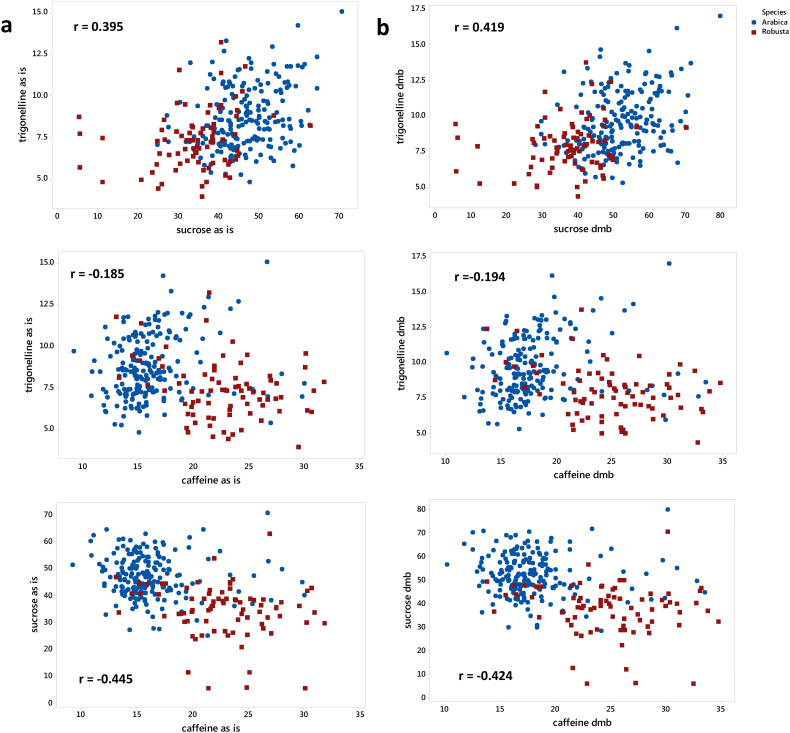
Correlation plots among sucrose, caffeine and trigonelline in single green coffee beans including Arabica and Robusta species. a) Values expressed on a wet (“as is”) basis or on b) dry weight basis, obtained by applying a HSI moisture calibration. Pearson correlation coefficients are indicated in the figure (*p* < 0.001 in all cases).

The presence of correlations among the three analysed coffee constituents was investigated, and a correlation was found among the compounds when expressing the data on an “as is” basis (please refer to Supplementary Figure 2). The correlation is statistically significant (*p* < 0.01) in every case, with a positive Pearson correlation observed for sucrose and trigonelline (i.e. 0.395). In contrast, negative correlations were observed for sucrose vs. caffeine has *r* = − 0.445, and caffeine vs. trigonelline (− 0.185). Previous research reported a negative correlation between caffeine and trigonelline in roasted coffee beans (Casal et al., 2000), however it was for bulk coffee and not investigating these compounds on single beans. In addition, trigonelline content dramatically changes due to roasting, as a function of the intensity of the roasting degree, thus a comparison cannot be made. The correlation among the three constituents analysed might be also influenced by the presence of different levels of moisture which can naturally fluctuate between the green coffee beans, especially when considering single coffee beans instead of batches. When concentrations were expressed on a dry matter basis, a slight negative but significant correlation was found between caffeine and trigonelline (*r* = − 0.194), and a stronger negative correlation was observed between sucrose and caffeine (*r* = − 0.424), while a weak significant positive correlation was observed between sucrose and trigonelline (*r* = 0.419, *n* = 277) (please refer to Supplementary Figure 2). These results are in accordance with Leroy et al. (2011), who described the absence of any significant correlation among these two coffee constituents on a wide range of coffee genotypes, over several years.

### 3.2 PLS regression for coffee constituents prediction based on HSI

**Fig. 1:**
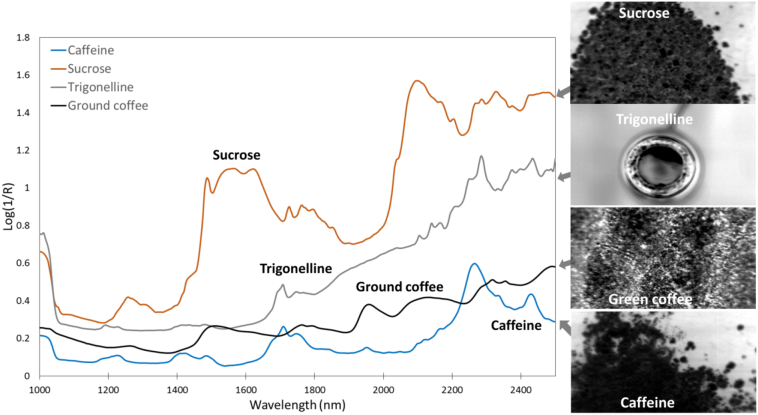
Average spectra obtained by HSI on samples of pure reference caffeine, sucrose and trigonelline, and example of a ground green coffee bean, showing also the absorbance images obtained from the hypercube at one spectral band ~ 1400 nm (right).

Trigonelline, sucrose and caffeine show characteristic absorption features in the NIR region (Fig. 1). While sucrose has significantly higher log(1/R) values, trigonelline and caffeine showed similar absorption bands. Caffeine main absorption peaks were at 1668, 2250 and 2425 nm. Trigonelline had similar absorption peaks at 1668 and 2269 nm. Sucrose had a characteristic absorption in the region 1460–1700 nm, and a second major peak observed around 2070 nm. The reflectance spectra of whole coffee beans show strong absorption features around 1430 nm, followed by two peaks at 1730 and 1760 nm, and another major peak at 1930 nm. Although the spectra are noisier in the upper wavelength region, another major absorption peak was observed around 2250 nm.

The peak around 1430 nm is related to the C—H stretch and C—H deformation vibration, whereas the region around 1800–1900 nm indicates the vibration of the second overtone of the carbonyl group, and the peak observed at 2250 relates to the –OH vibration. The absorptions at 1730 and 1760 nm are related to lipids, as they are attributed to the –CH_3_ and –CH_2_ overtone, respectively (Huck, Guggenbichler, & Bonn, 2005).

Table 2 reports the performance of PLS regression models for the prediction of sucrose, caffeine and trigonelline in single green coffee beans using several pre-processing techniques. Two separate models were built: one on “as is” basis and a second built on a predicted dry weight basis. Generally, the two approaches resulted in a similar prediction performance, with slightly higher errors for the dry matter basis groups. The best caffeine and trigonelline models produced R^2^ values of R_c_^2^ = 0.85 and 0.82, respectively. The RMSECV value for caffeine was 1.9 mg g^− 1^, and 1.0 mg g^− 1^ for trigonelline. The sucrose model performed considerably worse, with R_c_^2^and R_cv_^2^ in the region of 0.65 and 0.5, respectively, and RMSECV of 7.25 mg g^− 1^ on an “as is” basis.

**Table 2:** Performance of the PLS regression models for sucrose, caffeine and trigonelline, for HSI quantification on single green coffee beans, with coffee constituents expressed on (a) “as is” basis or (b) dry matter basis.

Compound	Treatment	LV	Calibration		Cross-validation	
			R^2^	RMSEC	R^2^	RMSECV
a. Reference data on “as is” basis						
Sucrose	log(1/R)	20	0.651	5.931	0.506	7.071
	SNV + 1st derivative	16	0.652	5.894	0.476	7.254
	**2nd der**	**12**	**0**.**647**	**5**.**912**	**0**.**462**	**7**.**250**
Caffeine	log(1/R)	13	0.666	2.647	0.628	2.799
	**SNV** + **1st der**	**16**	**0**.**851**	**1**.**635**	**0**.**793**	**1**.**929**
	2nd der	10	0.782	2.068	0.724	2.333
Trigonelline	log(1/R)	23	0.761	0.958	0.665	1.135
	**SNV** + **1st der**	**25**	**0**.**820**	**0**.**809**	**0**.**713**	**1**.**022**
	2nd der	17	0.823	0.794	0.703	1.034

b. Reference data on dry weight basis						
Sucrose	log(1/R)	20	0.653	6.489	0.509	7.751
	SNV + 1st der	16	0.564	7.485	0.482	8.181
	**2nd der**	**15**	**0**.**701**	**6**.**119**	**0**.**526**	**7**.**722**
Caffeine	log(1/R)	20	0.799	2.107	0.729	2.451
	**SNV** + **1st der**	**16**	**0**.**851**	**1**.**843**	**0**.**792**	**2**.**177**
	2nd der	11	0.797	2.159	0.726	2.513
Trigonelline	log(1/R)	25	0.795	0.979	0.689	1.208
	**SNV** + **1st der**	**25**	**0**.**833**	**0**.**860**	**0**.**733**	**1**.**090**
	2nd der	19	0.842	0.843	0.709	1.146

LV = Latent Variable, or Principal Component. RMSEC = root mean square error of calibration. RMSECV = root mean square error of cross-validation. The errors are expressed as mg g^− 1^ coffee beans. Bold indicates the prediction models with the best performance; preference was given to models with spectral pre-treatments applied and use of fewer LVs without significant reduction in prediction performance.

On the whole, the model built on the second derivative of the spectra was deemed to be the best sucrose model because it uses the fewest latent variables (LVs) with little effect on the overall performance, and utilises a spectral pre-treatment method. These factors make this model the most robust by avoiding overfitting, while also helping to reduce the effect of scattering relative to the untreated log(1/R) model.

According to Shenk and Westerhaus (1996), calibrations with R^2^ values between 0.50 and 0.69 can be used to separate into low, medium and high values, while R^2^ of 0.70–0.89 can be considered as good enough for quantification purposes. Based on the obtained RMSECV values, the models can therefore be considered as good for caffeine and trigonelline, but poor for sucrose quantification, although this model could still be used for screening purposes. As a proportion of the range, the cross-validation error was 11.1, 8.5 and 9.2% for sucrose, caffeine and trigonelline (“as is”), respectively. When expressing the values on a dry matter basis, this proportion was 10.4, 8.8 and 8.6%, respectively.

The quality of the calibrations presented can be evaluated using the Ratio of Performance Deviation (RPD), which is calculated as the ratio between the standard deviation and the standard error of cross-validation (Fearn, 2002, Williams and Sobering, 1993). RPD is dimensionless and higher values correspond to better analytical performance. In the present experiment, the lowest performance was obtained for sucrose, with RPD of 1.4 (“as is” basis) and 1.5 (dwb). For caffeine and trigonelline, the RPD values were very similar between the “as is” basis and dry matter basis; caffeine had RPD = 2.7 and trigonelline RPD = 2.0. For trigonelline, despite the significantly lower prediction error (i.e. almost half the RMSECV of caffeine), the higher RPD value was explained by the more limited range observed for trigonelline content. Despite the low prediction ability of such calibration, previous literature for other products still suggested the application of PLS calibrations with RPD values of 1.3–1.6 for in-line analysis, as it might be useful for breeding programmes to select particularly higher or lower values in a population (De Marchi, Riovanto, Penasa, & Cassandro, 2012).

**Fig. 2:**
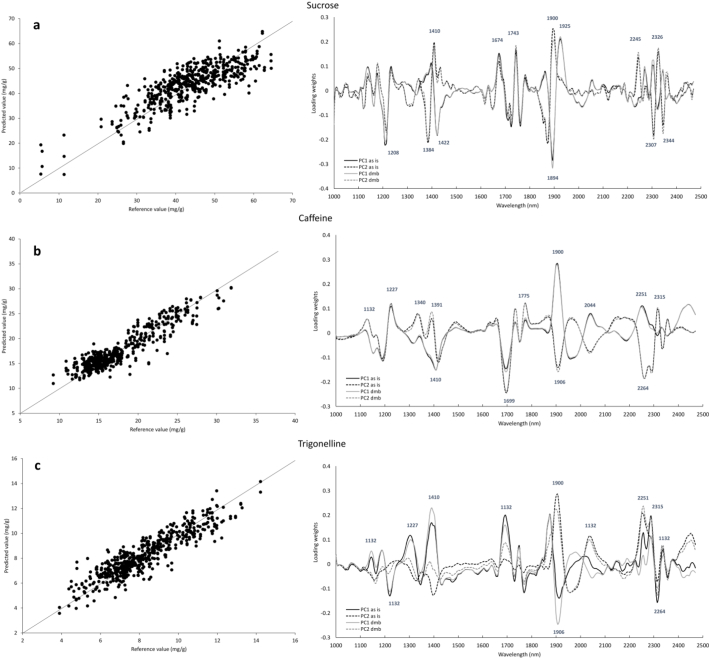
Predicted versus measured values of (a) sucrose, (b) caffeine and (c) trigonelline in single green coffee beans for the best PLSR models, expressing the compound concentration as mg g^− 1^ coffee (“as is” basis) (left). Loading weights for the same constituents, showing the models built on the reference measurements on “as is” basis or on a predicted dry weight basis (right).

Fig. 2 shows the predicted versus measured plots of the best PLS models for sucrose, caffeine and trigonelline prediction in single green coffee beans, and also reports the loading weights of the first two Principal Components for the three compounds.

Previous studies reported that the second derivative pre-treatment was less effective than the first derivative in terms of PLS regression models for chemical components such as caffeine, theobromine and theophylline (Huck et al., 2005). The PLS regression model based on NIRS reported by these authors for caffeine had R_c_^2^ value of 0.86 and R_cv_^2^ = 0.82. However, the authors predicted caffeine content on liquid coffee extracts having caffeine concentrations from 0.1 to 4.1%, thereby enhancing the capability of NIRS by removing the influence of other non-extractable compounds. Despite this, the prediction error was 3.4 and 4.0 mg g^− 1^ for the calibration and validation datasets, respectively.

As previously described, the application of HSI for quantitative prediction of chemical composition is currently very limited for coffee. Nonetheless, a recent paper by Zhang, Jiang, Liu, and He (2017) reported on the application of HSI on roasted coffee beans to investigate their caffeine content. The authors worked in the NIR region 874–1734 nm, but instead of using single coffee beans the reference caffeine analysis was carried out on a small batch of 20 coffee beans, ground and analysed by a spectrophotometric method. Despite 10 times lower caffeine concentrations between their study and ours (ranges 4–11 mg g^− 1^, 12–15 mg g^− 1^ and 10–23.5 mg g^− 1^ were reported in the previous study), the authors claim a good prediction performance for caffeine, i.e. R_c_^2^ = 0.897 and R_cv_^2^ = 0.834, and prediction errors of RMSEC = 111.3 and RMSECV = 142.1 μg g^−1^.

Generally, larger prediction errors typically expected for calibrations obtained by HSI compared to traditional NIRS instruments – and especially for ground material and liquid coffee extract or coffee brew – are attributed to the following factors: the sample is less homogenous than ground coffee; the sample presentation is less uniform and the illumination conditions are different; the sample size is typically smaller for HSI, which influences both the spectral acquisition and the reference analysis. These conditions therefore tend to result in poorer prediction models.

Despite these considerations, the performance of the HSI models compares favourably with the existing literature on NIR spectroscopy. For instance, Pizarro et al. (2007) reported an R^2^ above 0.99 for caffeine calibration in roast and ground coffee. Fox et al. (2013) investigated the use of FT-NIRS (~ 830–2500 nm) for caffeine quantification in whole and ground coffee, also testing single coffee beans, taking 25 beans from five batches. The prediction performances reported were generally better than our calibration model, but the authors also included a decaffeinated batch in their samples, which could have greatly influenced the model. In addition, it should be noted that their instrument has different sample presentation and illumination, but there are limitations related to the capability to implement it on an industrial scale for rapid and on-line measurement of single coffee beans. It was shown that caffeine prediction in roasted single coffee beans has worse performance compared to ground coffee or unground bulk coffee. The R^2^ reported for single beans without considering decaffeinated samples (i.e. using 4 batches of roasted coffee) was 0.93, with a cross-validation R^2^ of 0.86. The RMSEC and RMSECV were 1.2 and 1.6 mg g^− 1^, respectively (Fox et al., 2013). However, despite the slightly worse calibration performance there are two main advantages to using HSI. Firstly, traditional NIR instruments do not provide any spatial information, and secondly, even if it is technically possibly to create calibrations on single coffee beans, applying such calibration on a considerable number of beans would be time-consuming. On the whole, it is difficult to directly compare the performance of HSI-based calibrations and NIR calibrations or other techniques, and it is not only the analytical accuracy that should be considered, but also the practical advantages offered by each technique.

As part of the current study, prediction models were additionally built on the datasets for Arabica-alone and Robusta-alone. No difference in prediction capability was observed between the coffee species (data not shown), but the separate models had worse performance compared to the general model shown in Table 2, with particularly low RMSECV compared to the RMSEC. In some cases, this effect is likely to be caused by the limited number of samples when only Robusta samples are used. The only exception observed was for caffeine prediction, for which the model built on the Robusta dataset (*n* = 140) resulted in a far better performance than the Arabica model (*n* = 410), but still did not reach the level of performance of the general model. This result is useful as a prediction model for both Arabica and Robusta species as it would allow the prediction of the coffee constituents studied, without the need to discriminate the coffee species and apply separate calibrations for screening the raw material. With regards to species discrimination, Caporaso, Whitworth, Grebby, et al. (2017) demonstrated 100% discrimination ability between Arabica and Robusta coffees, both as green and roasted coffee beans, using HSI and Linear Discriminant Analysis (LDA). Moreover, unsupervised methods have also been previously applied using HSI for coffee species classification (Calvini et al., 2015). More limited research has been applied using HSI for quantitative prediction of food chemical composition. In this case, the prediction performance strongly depends on the compound investigated.

In spite of the slightly lower prediction performance frequently obtained by HSI compared to NIRS, and the expected lower prediction error when analysing single seeds, the HSI calibrations herein reported offer the advantage of screening single coffee beans without any preparation step, and detecting beans with extreme values in a rapid way.

Table 3 reports the performance of PLS regression models built on a reduced number of spectral variables, which were selected according to the regression coefficients of the full spectra models. The selection of a few important bands enables the compositional prediction using multispectral imaging systems, which have lower instrumentation costs and might permit faster data processing due to the reduced computation capacity. The multi-band models utilised between 5 and 10 wavelengths and 3–9 latent variables (LV). The model performance did not decrease appreciably in reducing the number of bands from 7 to 10 to 5 bands, with both giving similar prediction error. Using 5 spectral bands, the best performance was observed for caffeine, which showed calibration and cross-validation R^2^ = 0.69 and cross-validation error 2.35 mg g^− 1^ (as is), with even better performance for the dmb model, resulting in cross-validation R^2^ = 0.76. The model using 9 wavebands had cross-validation R^2^ = 0.80 and RMSECV below 2.1 mg g^− 1^. This error was even lower than the one obtained using the full spectra, which is probably due to the reduction of some unimportant bands and an added emphasis on the more diagnostic absorbance features of caffeine at specific wavelengths. In contrast, a dramatic decrease in the model performance was obtained for trigonelline prediction, especially when the compound content was expressed on dry matter basis. These results indicate that the band reduction causes worst prediction performance for sucrose and trigonelline, but still acceptable prediction ability for caffeine, suggesting its use for screening purposes.

**Table 3:** Wavelength selection for HSI prediction of sucrose, caffeine and trigonelline, expressed on (a) “as is” basis, or (b) dry matter basis.

	Pre-treatment	Band number	LV	Calibration		Cross-validation		RPD
				R^2^	RMSECV	R^2^	RMSECV	
a)								
Sucrose	2nd der.	7	3	0.416	7.604	0.402	7.708	1.32
		5	3	0.414	7.617	0.406	7.689	1.33
Caffeine	SNV + 1st der.	7	5	0.702	2.314	0.694	2.349	2.00
		5	4	0.693	2.346	0.688	2.372	1.98
Trigonelline	SNV + 1st der.	9	5	0.402	1.475	0.392	1.490	1.34
		5	4	0.399	1.478	0.391	1.490	1.34

b)								
Sucrose	2nd der.	10	4	0.443	7.820	0.422	7.971	1.44
		5	2	0.405	8.085	0.393	8.176	1.41
Caffeine	SNV + 1st der.	9	9	0.813	2.018	0.801	2.082	2.45
		5	4	0.762	2.276	0.755	2.308	2.21
Trigonelline	SNV + 1st der.	10	7	0.277	1.790	0.256	1.817	1.21
		5	4	0.280	1.719	0.270	1.735	1.27

LV = latent variable. RPD = ratio to performance deviation (ratio between standard deviation of the reference measurements over the RMSECV). The errors are expressed as mg g^− 1^ coffee beans.

### 3.3 Application of the calibrations and visualisation of “chemical images”

**Fig. 3:**
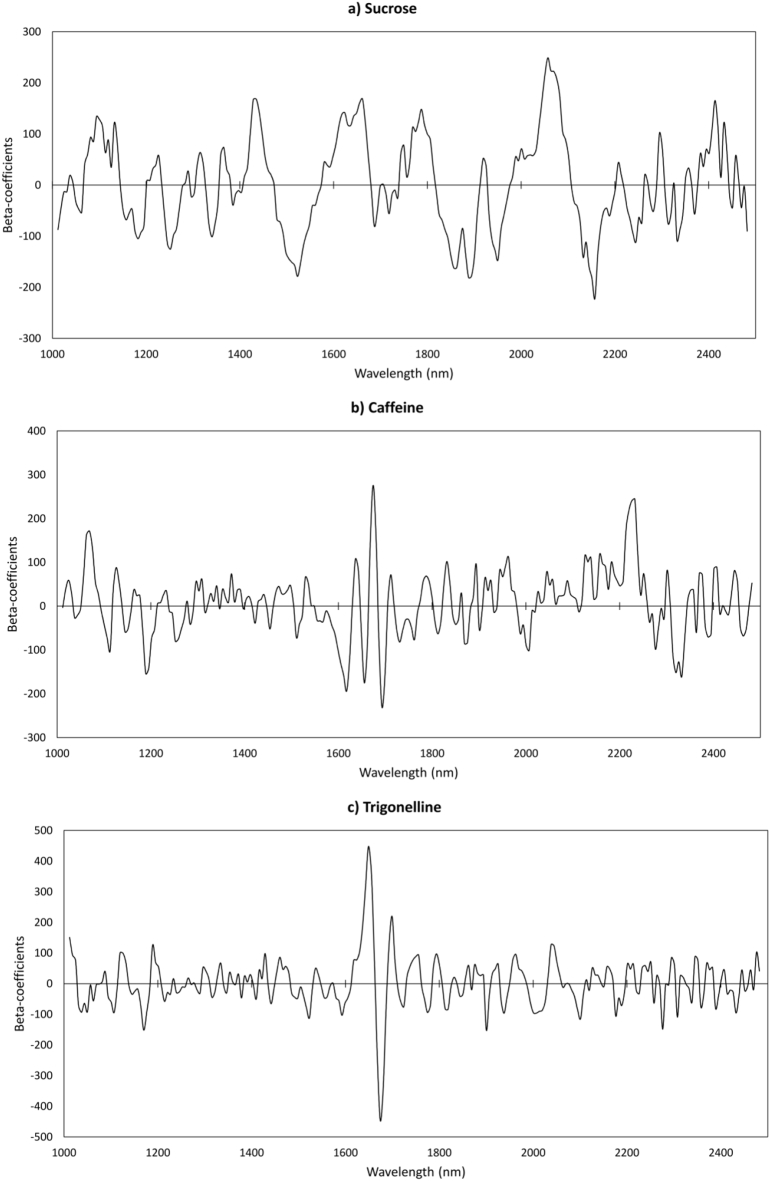
Regression coefficients for the PLSR models built on single green coffee beans, for the prediction of the three coffee constituents. Pre-treatments applied: sucrose, 2nd derivative; caffeine and trigonelline, SNV + 1st derivative.

The best prediction models for the three compounds analysed were selected and the beta-regression coefficients (shown in Fig. 3 for each of the coffee constituent predicted) were applied to the treated hypercubes after applying image segmentation and spectral pre-processing, so that a “chemical image” was generated to reveal the distribution of compounds at a single pixel level.

**Fig. 4:**
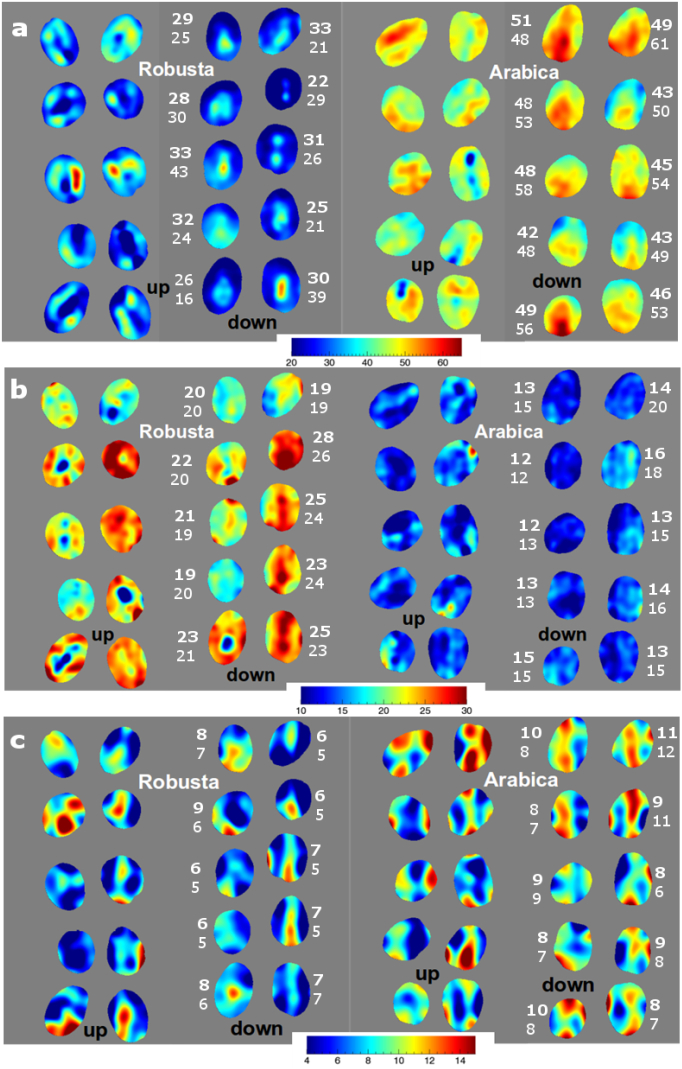
Application of the calibrations to visualise (a) sucrose, (b) caffeine and (c) trigonelline in a batch of Robusta and Arabica green coffee beans, at a single pixel. Numbers in the figure indicate the concentration of each compound, expressed as mg g^− 1^ coffee. Numbers in bold indicate the predicted compound concentration, followed by the reference measurement for each coffee bean.

The application of the best PLS calibrations for sucrose, caffeine and trigonelline are shown in Fig. 4, using a batch of Robusta coffee from Vietnam and an Arabica coffee sample from Kenya. Each sample was scanned twice to acquire both sides of the coffee bean (“up” and “down”), and they are shown separately. The numbers indicated in bold represent the HSI prediction for each bean, while the other number for each bean indicates the content measured by the reference HPLC-MS analysis, expressed as mg g^− 1^ ground coffee. Very few differences were observed depending on the coffee bean position. Some minor effects observed within single coffee beans can be attributed to both actual chemical differences across the sample, but also to topographical differences, e.g. the midline crease that corresponds to the mesenteric root. This portion of the bean was excluded from the calibration stage as the absorbance values might have influenced the computation of a representative average spectrum for each bean.

**Supplementary Figure 3:**
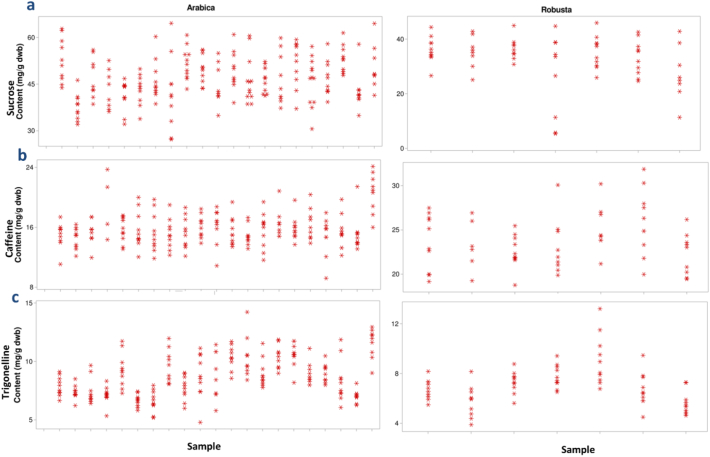
Single bean variability within batches of Arabica (left column) and Robusta (right column) green coffee beans from several origins, with respect to the content of a) sucrose, b) caffeine and c) trigonelline. Each column in the graph represents a single batch, while each symbol is an individual coffee bean analysed by HPLC-MS. The analysis was done by duplicate injection of the extract.

Once the calibration is applied, obvious differences in the sucrose and caffeine content are visible between Arabica and Robusta coffee beans. Arabica coffee beans had significantly lower caffeine content, with a more uniform distribution compared to the Robusta beans. For Robusta, variability was observed within and between coffee beans (please refer to Supplementary Figure 3 for further information), which is possibly due to differences in the total caffeine as affected by the maturity degree at collection or uneven post-harvest processing and drying. When visualising the predicted trigonelline content, little difference between the species was apparent. Robusta samples generally had lower predicted trigonelline levels, with a few samples showing higher content. Arabica samples show interesting differences within the same coffee beans, without a clear general pattern. This effect might be due to physiological reasons, possibly related to the migration during the post-harvest process over the external layers of the beans, or to the cell compartmentalisation. There is also the possibility that some of the observed variability across a single coffee bean might be due to noise in the spectra. Either way, further research is needed to verify whether genuine differences do exist. This would primarily be dependent on the availability of analytical techniques that would enable the acquisition of reference data for specific locations on individual beans.

HSI calibrations built on single objects can be applied on an individual pixel basis, or to average spectra for single objects so that the mean predicted values are obtained, as previously reported for wheat kernels (Caporaso, Whitworth, & Fisk, 2017). In this way, HSI is able to provide information on a single coffee bean basis and therefore an on-line system for rapid scanning could potentially be developed based on the proposed approach. Accordingly, it is possible to visualise target chemical compounds on a single bean basis and obtain the distribution within single beans, once a calibration is applied to individual pixels. Such detailed information is not limited just to the food and beverage sector, but could be also useful for studies in the botanical, plant physiology and plant genetic fields.

## 4 Conclusions

Hyperspectral imaging was applied to single green coffee beans for the purpose of exploring the potential to quantify chemical constituents non-destructively without any sample pre-treatment, and reveal the concentrations on a single coffee bean basis. HSI was shown to be effective for the non-destructive and rapid analysis of sucrose, caffeine and trigonelline in green coffee beans, which are compounds that affect the final flavour of coffee. The devised method allows the rapid prediction of coffee constituents not just for batch measurements, which could be performed with traditional NIR spectroscopy, but simultaneously on multiple individual coffee beans. The RMSECV for sucrose, caffeine and trigonelline was 7.3 mg g^− 1^, 1.9 mg g^− 1^ and 1.0 mg g^− 1^ (“as is”), respectively, and therefore acceptable for both screening (sucrose) and quantification (caffeine and trigonelline).

This is the first reported application of HSI for the prediction of sucrose, caffeine and trigonelline on a single coffee bean basis. Additionally, this research provides information on single green coffee bean variability and is the largest study on HSI calibration for this commodity. Despite lower prediction accuracy compared to traditional analytical techniques, the advantages offered by hyperspectral chemical imaging are of particular interest for practical applications, such as the screening of single coffee beans, both at the research level and for industry. As demonstrated here for the first time, HSI is a useful tool for exploring the natural variability of sucrose, caffeine and trigonelline at a single coffee bean level. Moreover, with additional development, HSI could be applied for the screening of single beans to be further analysed using other techniques, such as in breeding programmes to select coffee beans with particular characteristics. Furthermore, the spatial distribution of the compounds was revealed across individual beans, which may be of interest to roasters, breeders or biologists.

While HSI is used here in conjunction with the classical PLS regression approach, future research will focus on exploring advanced HSI data analysis techniques, including non-linear prediction models. However, given the increased complexity of such techniques, it is likely that methods for reducing data redundancy would need to be applied before they can be implemented efficiently and effectively for HSI analysis of food commodities.

The following are the supplementary data related to this article.
